# Positive Impact of Professionalism on the Perception of Global Well-Being: A Study in Healthcare Professionals Starting Their First Working Experience in Peruvian Rural Areas

**DOI:** 10.3389/fpubh.2020.575774

**Published:** 2020-12-16

**Authors:** Humberto López-Morales, Edgar Rivera-Diaz, Andrew Ore-Zuñiga, Angel Vera-Portilla, Montserrat San-Martín, Roberto C. Delgado Bolton, Luis Vivanco

**Affiliations:** ^1^Faculty of Medicine, National University of San Agustin, Arequipa, Peru; ^2^Faculty of Social Sciences of Melilla, University of Granada, Melilla, Spain; ^3^Department of Diagnostic Imaging (Radiology) and Nuclear Medicine, University Hospital San Pedro, Logroño, Spain; ^4^Platform of Bioethics and Medical Education, Centre for Biomedical Research of La Rioja (CIBIR), Logroño, Spain; ^5^National Centre of Documentation on Bioethics, Rioja Health Foundation, Logroño, Spain

**Keywords:** professionalism, empathy, subjective well-being, interdisciplinary health teams, learning, rural health services, primary health care

## Abstract

**Introduction:** In Peru, recently graduated physicians and nurses who are willing to start working in the public healthcare system, first have to work in their newly acquired profession in the programme denominated “*Servicio Rural Urbano y Marginal de Salud*” (SERUMS). The SERUMS programme is a 1-year contract in rural areas of the country. The aim of this study was to confirm the following hypothesis: the development of abilities associated to professionalism has a positive effect on the perception of global well-being in the professionals beginning SERUMS.

**Material and methods:** In the study two cohorts of medical and nursing professionals that started SERUMS in 2017 and 2019 were included. The perception of global well-being and general health condition were measured with the Scale of Life Satisfaction (SWLS) and the General Health Questionnaire (GHQ-28), respectively. Professionalism was measured using Jefferson's scales of empathy (JSE), teamwork (JSAPNC), and lifelong learning (JeffSPLL). An analysis in phases using the R language was applied to develop a multiple regression model that would explain the lineal relationship between the global perception of well-being and the studied variables.

**Results:** The study sample included 303 professionals (108 men and 195 women) with a mean age of 26 years, ranging from 22 to 39 years (*SD* = 4). Based on their profession, 230 were medical doctors and 67 were nurses. The multiple regression model evidenced that age (*p* < 0.001), social dysfunction (*p* < 0.001), severe depression (*p* < 0.001), and inter-professional collaborative work abilities (*p* < 0.001) explain 38% of the variability in the global perception of well-being. Moreover, a second model explained 44% of the variability in the inter-professional collaborative work abilities based on a lineal relationship with empathy (*p* < 0.001), lifelong learning (*p* < 0.001), and future professional orientation (*p* = 0.01). Both models complied with the necessary conditions for statistic inference and showed large effect sizes.

**Conclusions:** These findings confirm that professionalism has an important role in improving the global well-being of the professionals initiating SERUMS. This influence is direct in the case of inter-professional collaborative work, whereas it is indirect in the case of empathy and lifelong learning.

## Introduction

In the field of healthcare, the transition from university to professional life is a stage of change usually accompanied by challenges and uncertainties, both personal and professional (1). Therefore, at this stage several authors recommend that these professionals have suitable accompaniment and supervision that facilitate a healthy adaptation to the world of work while guaranteeing proper patient care (2, 3).

In Latin America, one's first work experience is usually done in national social health service programmes. The first country to establish this type of programme was Mexico in 1936 to serve the rural communities in its territory (4). Over the years, many other countries in the region have incorporated similar programmes to meet the primary care needs of their health systems. Although there are differences between the countries, these programmes usually have three elements in common: (i) they are aimed at recently graduated professionals; (ii) they have a certain compulsory nature; and (iii) they are carried out in rural areas. However, in the last 20 years, the effectiveness of these programmes has been questioned in various international forums, as a consequence of significant deficiencies: limited training value, low monetary remuneration, lack of supervision, little or poor preparation of the workers to carry out their tasks in rural areas, high accident rates, and a scarcity of logistical resources and facilities in the areas where this service is performed (5–9).

The social medical service was initially introduced in Peru in 1972 under the name of “*Servicio Civil de Graduandos*” (SECIGRA). SECIGRA was created as a compulsory unpaid service that medical students had to do as a final requirement for granting their degree. In 1980, SECIGRA gave way to the “*Servicio Rural Urbano y Marginal de Salud*” (SERUMS). Unlike the previous one, SERUMS consisted of a payed professional service that was performed by recently graduated physicians in rural and marginal areas of the country to cover primary care needs. Currently, SERUMS includes professionals from other health disciplines as well as medicine. The service, which is coordinated by the Ministry of Health, is governed by the Regulations of Law No. 23330 of 1997 (10). Maybe because SERUMS is a requirement for the subsequent access to medical specialization programmes in the country, because of the monetary income that comes with it, because it is a requirement for accessing the public health system, or because having done it is positively valued in the private sector, very few professionals do not participate in this programme. The important deficiencies that SERUMS presented in the past years have been widely documented in several studies (7–9, 11). In some cases, these deficiencies were such that they led to the loss of the lives of some of the young professionals who worked in this service (12, 13).

However, recent studies have warned of other aspects that influence the deterioration of the health and well-being of the professionals who carry out SERUMS but that are not directly associated with the programme itself. In 2011, a study in young recently graduated physicians who were starting SERUMS warned of a significant deterioration of their state of health and well-being (14). The uncertainty that characterizes the start of this new stage, especially under the aforementioned conditions, may be one of the reasons for this phenomenon. Another cause, no less important, can be found in the stage immediately before starting SERUMS. In Peru, medical and nursing students must complete a year of “medical internship.” This internship consists of a 12 month immersive clinical-training experience in some of the clinical services of public or private hospital institutions (for medical students) and in hospital and primary care services of the Public Health System (for nursing students). Recent studies have warned of a high level of psychological and emotional abuse and harassment among students who complete a rotating internship, especially in public hospitals (15–19). The severity of this situation is greater if one takes into account that, in many cases, those who carry out this harassment are the residents or attending physicians who are in charge of their training (19). In addition, in one of these studies, a worrying deterioration in the ethical and professional conduct of these students was observed as a consequence of the educational deficiencies of this period of clinical immersion (20). Finally, in one of the most recent studies done in Peru with medical and nursing students, an important difference has been reported in the development of inter-professional collaborative work skills among medical and nursing students in the last years of their degrees (21). This is a very significant fact, all the more so if one takes into account that, in SERUMS, both groups of professionals must carry out most of their work in a joint and coordinated manner and without any type of professional supervision.

The reality described above has little to do with the paradigm of modern medicine, oriented toward increasing professionalism. This professionalism, in the field of health, has been defined as the set of skills and values that characterize both the essence of humanity in dealing with patients and the search for professional excellence (22, 23). Within this framework, three professional competences have been described as specific components of such professionalism: empathy in dealing with patients, inter-professional collaborative work skills, and lifelong medical learning skills (24). There is a growing scientific literature that recognizes the important value that these three competences have in caring for the health and the emotional well-being of health professionals (25–27), in obtaining positive clinical results (28, 29), and for patient care (30, 31).

Based on the evidence above, this study was designed with the purpose of confirming the following hypothesis: that the development of skills associated with professionalism has a positive effect on the overall perception of well-being of health professionals who start SERUMS. If so, professionalism would offer healthcare professionals a series of personal and professional resources that are useful when faced with shortcomings in working conditions in their future workplace. This benefit should be reflected in a better global perception of well-being at this stage of their lives. Accordingly, there were four research objectives that were set during this study: (i) measure the general state of health and the global perception of well-being of a sample of health professionals starting SERUMS; (ii) measure empathic orientation in dealing with patients, collaborative inter-professional work skills and lifelong learning skills; (iii) identify which of these three elements have a positive effect on the overall perception of well-being of these professionals; and (iv) characterize the factors that influence the development of these elements.

## Materials and Methods

### Participants

Two cohorts of medical and nursing professionals participated in the study, who responded in April 2017 and 2019 to the call to fill vacancies of the *Gerencia Regional de Salud de Arequipa* (GERSA), the body responsible for coordinating SERUMS in the eight provinces that make up the Region of Arequipa. Professionals from disciplines other than medicine or nursing, who also responded to these calls, were not included in the study.

### Main Measures

The global perception of well-being was measured using a Spanish version of the Scale of Life Satisfaction (SWLS). The SWLS measures the degree of global life satisfaction of the individual (32). SWLS is a unifactorial scale with five items. The scoring of the Spanish version of the SWLS used in this study (33) is slightly different to the original one developed in English by Diener et al. (32). Each item of the Spanish version is scored with a 5-point Likert scale ranging from 1 (strongly disagree) to 5 (strongly agree), while in the English version each item is scored with a 7-point Likert scale. The Spanish version used in this study has been tested in adolescents, adults and healthcare professionals showing a high reliability (27, 33, 34). Higher scores are indicative of a better overall perception of well-being (subjective well-being).

The general state of health was measured with the General Health Questionnaire (GHQ-28). The GHQ-28 measures the individual's mental health by detecting recently emerging problems (35, 36). The 28 items in GHQ-28 are grouped into four areas composed by seven items: “somatic symptoms”; “anxiety and insomnia”; “social dysfunction”; and “severe depression.” Each item is scored in a 4-point Likert scale. The calculation of the general state of mental health comes from the global score achieved in all areas. A high score is associated with a recent deterioration of mental health in the areas studied.

Empathy was measured with the healthcare professional version of the Jefferson Scale of Empathy (JSE-HP). The JSE measures the empathic orientation of the health professional in their treatment of the patient (37). The 20 items in the JSE are grouped into three factors: “perspective taking,” “compassionate care,” and “walking in the patient's shoes.” Each item is scored in a 7-point Likert scale ranging from 1 (strongly disagree) to 7 (strongly agree). A higher score is associated with a greater empathic orientation in dealing with the patient.

Inter-professional collaboration was measured using the Jefferson Scale of Attitudes toward Physician-Nurse Collaboration (JSAPNC). The JSAPNC evaluates the skills for collaborative inter-professional work among medical and nursing personnel from four factors: “collaboration and shared education,” which refers to the global perception of inter-professional work, “caring as opposed to curing,” which consists of the ability to distinguish between the professional fields of action that characterize nursing medicine, “autonomy of the nurse,” and “authority of the doctor,” each referring to the perception of the specific professional field of medicine and nursing within an interdisciplinary team (38). Each of the 15 items of the JSAPNC is scored in a 4-point Likert scale ranging from 1 (strongly disagree) to 4 (strongly agree).

Lifelong learning was measured using the Jefferson Scale of Physicians Lifelong learning (JeffSPLL). The JeffSPLL measures the skills shown by the healthcare professional in searching for information, self-motivation, and taking advantage of learning opportunities (39). The 14 items of the JeffSPLL are distributed among three factors: “beliefs and motivations in learning,” “attention to learning opportunities” and “skills in the search for information.” Each item is scored in a 4-point Likert scale ranging from 1 (strongly disagree) to 4 (strongly agree).

The aforementioned instruments were accompanied by a socio-demographic form in which the following information was collected: gender, discipline (medicine or nursing), age, professional field they aimed at specializing in the future (primary care or specialities other than primary care), and the province where they would perform the SERUMS.

### Procedures

In the week before moving to their designated workplaces, healthcare professionals received an informative session in Arequipa city organized by GERSA. This session is currently performed at the beginning of April. In 2017 and 2019, at the end of this session attendees received an informative talk about the study. Two external researchers (HLM and AOZ) presented the talk. They distributed among those attendees that accepted to participate in the study a questionnaire containing the scales and a sociodemographic form inside a sealed envelope. Once the questionnaires were completed, the participants returned them in their sealed envelopes. The information in the questionnaires collected in both activities (2017 and 2019) was used to create a database that was later used in the statistical analysis.

The participation of health professionals was voluntary, confidential, anonymous and secret. Furthermore, all participants signed a written informed consent. The study had the approval of an independent ethical committee (*Comité de Ética de Investigación Cl*í*nica de La Rioja - CEICLAR*) and the approval of the *Dirección Regional de Salud de Arequipa*.

### Statistical Analysis

Only the questionaires with fully completed scores in all their items were included into the statistical analysis. The reliability or internal consistency of the psychometric instruments used was measured by calculating Cronbach's alpha coefficient. Values higher than 0.70 were considered satisfactory.

A multiple linear regression analysis was performed in which the measure of global perception of well-being (life satisfaction) was treated as an explained variable (dependent), while all the others were treated as possible explanatory variables (independent). Thanks to this analysis, it was possible to create a model that enabled the identification of variables that acted as influencing factors for subjective well-being. Later, another multiple linear regression analysis was performed to identify those variables that acted as influencing factors for the components of medical professionalism that appeared as explanatory variables for subjective well-being. In both analyses, whether the models obtained met the necessary conditions for statistical inference was studied. That is, normality, zero mean, constant variance and uncorrelatedness of the residuals, in addition to linearity and absence of multi-collinearity. Finally, in order to quantify the degree of practical significance of the findings observed in each model, the effect size (Cohen's *f*^2^) was calculated. An effect size equal to 0.02 was interpreted as small, equal to 0.15 was interpreted as medium, and equal to 0.35 was interpreted as large (40).

All analyses were done in the R language and programming environment for statistical and graphical analysis, version 3.5.2 for Windows and with the help of the statistical analysis packages *nortest* (41), *ApaTables* (42), and *multilevel* (43).

## Results

Of the 397 medical and nursing professionals who attended the allocation of places, 303 agreed to participate in the study (76% overall response rate). Of these, 137 were from the 2017 cohort and 166 from the 2019 cohort. Concerning gender, the sample included 108 men and 195 women. Regarding the discipline, 230 professionals were medics and 67 were nurses. The mean age was 26 years (*SD* = 4), with an age range from 22 to 39 years.

The first two objectives of this study were the measurement of the general state of health and the global perception of well-being (subjective well-being) (Objective 1); and the measurement of three specific components of professionalism: empathy, inter-professional collaboration and learning (Objective 2). A preliminary comparative analysis was performed for both cohorts of health professionals for each of the variables studied. As no statistically significant differences were found between the two groups, these were treated in subsequent analyses as a single sample. In this sample, the range of Cronbach's alpha coefficients was from 0.80 to 0.94. A summary of each coefficient, as well as the distribution of the frequencies achieved for each instrument is shown in Table 1.

**Table 1:** Descriptive analysis and psychometric reliability of the scale of life satisfaction, general state of health, empathy, inter-professional collaboration, and lifelong learning.

**Scales**	***n***	**PR**	**AR**	**Mdn**	**M (*SD*)**	**Reliability**
SWLS	234	5–25	5–25	18	18 (4)	0.83
GHQ-28	234	0–84	0–78	19	21 (12)	0.93
Somatic symptoms	234	0–21	0–18	6	6 (4)	0.83
Anxiety and insomnia	234	0–21	0–21	6	6 (4)	0.89
Social dysfunction	234	0–21	0–21	6	6 (3)	0.80
Severe depression	234	0–21	0–21	0	2 (4)	0.94
JSE	237	20–140	67–136	113	111 (14)	0.84
JSAPNC	236	15–60	29–60	46	47 (7)	0.82
JeffSPLL	236	14–56	32–56	47	47 (5)	0.83

*SWLS, Scale of Life Satisfaction; GHQ-28, General Health Questionnaire; JSE, Jefferson Scale of Empathy; JSAPNC, Jefferson Scale of Attitudes toward Physician-Nurse Collaboration; JeffSPLL, Jefferson Scale of Physicians Lifelong learning; n, fully completed questionnaires; PR, possible range; AR, actual range; Mdn, median; M, mean; SD, standard deviation*.

**Figure 1:**
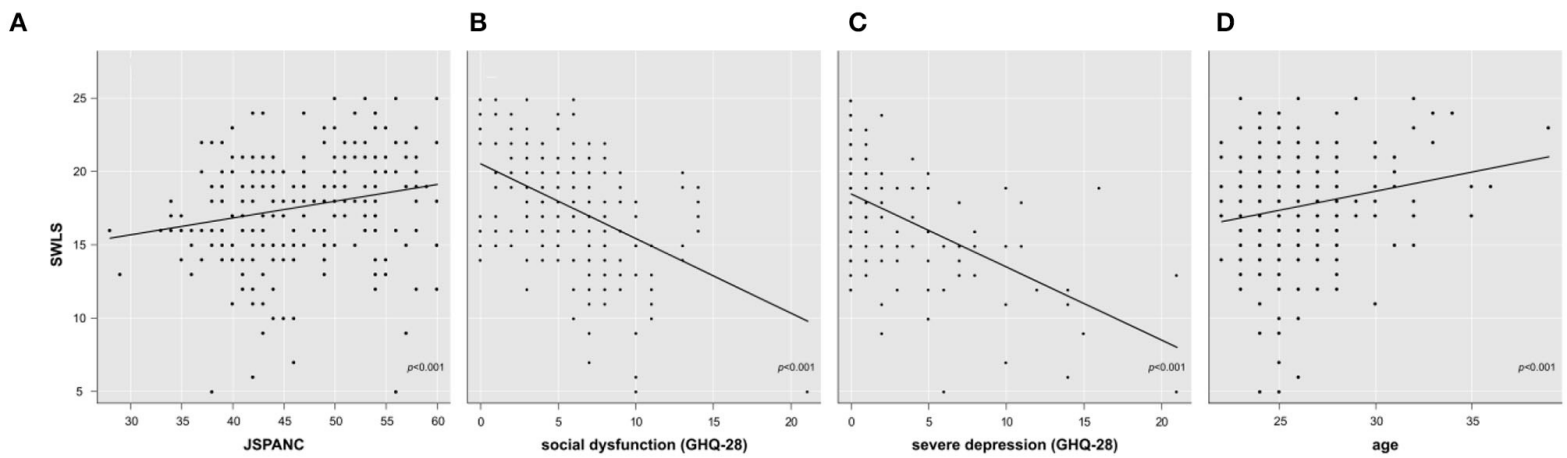
Variation in subjective well-being scores as a function of inter-professional collaboration skills **(A)**, social dysfunction **(B)**, severe depression **(C)**, and age **(D)**. SWLS, Scale of Life Satisfaction; GHQ-28, General Health Questionnaire; JSAPNC, Jefferson Scale of Attitudes toward Physician-Nurse Collaboration.

The next objective of this study (Objective 3) was to determine whether if professionalism, through any of the measured components, had a positive effect on the overall perception of well-being. Multiple linear regression analysis allowed the creation of a model explaining 35% of the variability of the global perception of well-being score (*R*^2^-adjusted = 0.34; *F*_(3,229)_ = 40.84; *p* < 0.001; Cohen's *f*^2^ = 0.54) based on the following variables: “inter-professional collaboration,” “general state of health” and “age.” In order to determine the specific areas of the GHQ-28 that were acting as explanatory variables, another analysis was performed allowing the creation of a model that explained 38% of the variability of the global perception of well-being score (*R*^2^-adjusted = 0.37; *F*_(4,228)_ = 34.5; *p* < 0.001; Cohen's *f*^2^ = 0.61). This model confirmed a linear relationship between the global perception of well-being and the following variables: “inter-professional collaboration,” “social dysfunction,” “severe depression” and “age” (Figure 1). A complete summary of this analysis is described in Table 2. Both models fulfilled the necessary conditions for statistical inference with a large effect size.

**Table 2:** Multiple linear regression model for global perception of well-being.

	**β**	***SE***	***t***	***p***	**VIF**
Inter-professional collaboration (JSAPNC)	+0.10	0.03	+3.51	<0.001	1.01
Social dysfunction (GHQ-28)	−0.28	0.07	−3.97	<0.001	1.40
Severe depression (GHQ-28)	−0.35	0.06	−5.77	<0.001	1.41
Age	+0.24	0.07	+3.55	<0.001	1.02

*JSAPNC, Jefferson Scale of Attitudes toward Physician-Nurse Collaboration; GHQ-28, General Health Questionnaire; β, beta coefficient; SE, standard error; t, t experimental; p, p-value; VIF, variance inflation factor*.

*R^2^-adjusted = 0.37; F_(4, 228)_ = 34.50; p < 0.001; Cohen's f^2^=0.61*.

**Figure 2:**
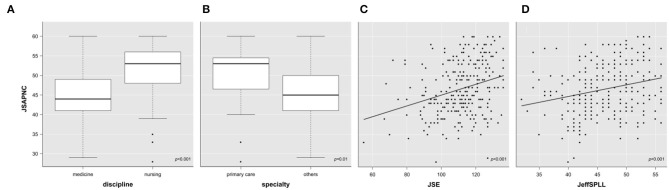
Variation in scores for inter-professional collaboration skills according to discipline **(A)**, speciality **(B)**, empathy **(C)**, and learning **(D)**. JSE, Jefferson Scale of Empathy; JSAPNC, Jefferson Scale of Attitudes toward Physician-Nurse Collaboration; JeffSPLL, Jefferson Scale of Physicians Lifelong learning.

The last objective (Objective 4) of this study was to characterize factors influencing the development of the components of professionalism that have a positive effect on subjective well-being. Of the three components measured, inter-professional collaboration appeared as one of the elements that positively influence the overall perception of well-being. Therefore, a second regression analysis was carried out to determine which of the variables studied influence the development of this competence. Thus, inter-professional collaboration was used as a dependent variable with the aim was of creating a model that would allow the identification of factors (independent variables) that influence the variation in that element. The analysis produced a model that explained 44% of the variability of the JSAPNC measurement (*R*^2^-adjusted = 0.43; *F*_(4,222)_ = 42.85; *p* < 0.001; Cohen's *f*^2^ = 0.77). The model included the variables: “discipline” (nursing) and “speciality” (other than primary care), “empathy,” and “learning” (Figure 2). All these variables showed a positive linear relationship, except for the speciality. A summary of this analysis is shown in Table 3. This second model also fulfilled the necessary conditions for statistical inference and presented a large effect size.

**Table 3:** Multiple linear regression model for inter-professional collaboration skills.

	**β**	***SE***	***t***	***p***	**VIF**
Empathy (JSE)	+0.09	0.02	+3.42	<0.001	1.07
Lifelong Learning (JeffSPLL)	+0.23	0.07	+3.38	<0.001	1.06
Discipline (nursing)	+8.10	0.82	+9.83	<0.001	1.09
Speciality (other than primary care)	−2.88	1.15	−2.50	0.01	1.08

*JSE, Jefferson Scale of Empathy; JeffSPLL, Jefferson Scale of Physicians Lifelong learning; β, beta coefficient; SE, standard error; t, t experimental; p, p-value; VIF, variance inflation factor*.

*R^2^-adjusted = 0.44; F_(4, 222)_ = 42.85; p < 0.001; Cohen's f^2^ = 0.77*.

## Discussion

The Cronbach's alpha coefficient values for all the scales used were ≥0.80, confirming the adequate psychometric properties of all the instruments. Furthermore, these values were similar to or higher than those described in the original versions (32–35, 37–39).

The main finding of this study was the experimental demonstration of the predictive role that professionalism has in the subjective perception of well-being in healthcare professionals starting SERUMS. This role was demonstrated directly in the case of inter-professional collaboration skills and indirectly in the case of empathy and lifelong medical learning skills.

For the inter-professional collaboration skills, the findings of this study confirm the role that these skills have as a positive influence on the global perception of well-being of recently graduated professionals. This finding is in line with those reported in previous studies describing a positive association between certain social skills, mainly oriented to the development of positive relationships with others, and indicators of psychological well-being (44) and occupational well-being (26, 45). In this regard, the JSAPNC is a tool designed for the measurement of specific skills oriented to collaborative inter-professional work in medicine and nursing. The tool evaluates the assessments made on shared education and the ability to recognize differences and complementarity between the two professional areas (38). It is to be expected that professionals presenting a greater development of these skills, and who, therefore, score higher in this tool, will have an easier time: (i) generating positive work environments, (ii) making better use of resources, (iii) adapting more quickly to new circumstances, and (iv) establishing channels of communication and personal and professional support with professionals from other healthcare areas in their new post. All these elements would, therefore, be highly valuable personal resources in their new work environment, despite the important differences and particularities that the rural environment may present. In addition to the aforementioned skills, general health status and age appeared as two other influencing factors in the perception of subjective well-being in recently graduated professionals starting SERUMS. The deterioration in the general state of health, measured using the GHQ-28, covers four main elements: somatisation, anxiety, social dysfunction and severe depression (35). Recent studies in nurses with years of professional experience (44, 45) and in medical students (46) have described an inverse association between these elements and life satisfaction. As in those publications, the findings in this study not only confirm this association but also provide experimental evidence of the predictive nature of the deterioration in social dysfunction and severe depression, two components of the general state of health measured by the GHQ-28, for the overall perception of well-being. In Peru, where the adaptation period between graduation and the start of rural medical service comprises only a few weeks, the physical, psychological and emotional deterioration due to the stress in the previous training stages can be an important cause of deterioration in the general state of health of the recently graduated professionals starting SERUMS (15, 16, 47). This, in view of the current findings, can have very harmful effects on the well-being of the professionals starting SERUMS. Furthermore, these findings coincide with other, also reported in Peru, showing a high prevalence of depression and alcohol abuse in physicians starting their first working experience in SERUMS (12). Finally, age appeared as a third influencing factor, in this case positive, on the perception of subjective well-being among these professionals. This finding is in line with the increase in emotional stability that accompanies age and which has been extensively studied (48–50). For this study, it is understandable that older professionals have greater confidence and personal resources than their younger colleagues when faced with the challenges that accompany this new stage of their personal life and the first of their working life.

Regarding the factors that influence the development of inter-professional collaboration skills, as well as discipline and professional direction (future speciality), empathy and lifelong learning skills appeared as two factors influencing the development of inter-professional work skills. Empathy, in the specific context of clinical encounters, has been defined as a predominantly cognitive (rather than affective or emotional) professional competence characterized by three elements: understanding (of patients' experiences and concerns); good and clear communication; and altruism (expressed in a compassionate attitude to care a person in need). It is demonstrated that an empathetic engagement based on the three above-mentioned elements not only protects from the negative effects caused by an intensive emotional involvement, but also creates a positive and more satisfactory working environment (26), even in adverse circumstances (27). On the other hand, lifelong learning is described as a component of both excellence and self-regulatory and accountable behavior to ensure quality of care. This ability involves an asset of self-initiated conduct and information-seeking skills that are activated in individuals with a sustained motivation to learn and the ability to recognize their own learning needs. There is a demonstrated association between lifelong learning and some indicators of occupational well-being such as motivation, professional accomplishments, career satisfaction, and professional commitment (39). Furthermore, in healthcare professionals, a greater development of these abilities is associated with a reduction in the workers' stress as consequence of having abilities to control work activities (51). As it is, these two elements, empathy and lifelong learning, appear as factors that indirectly influence the professional's improvement in subjective well-being for those starting SERUMS. Previous studies have shown a certain amount of overlap between empathy and inter-professional collaboration skills in medical students (52). Similarly, a positive association has been reported between inter-professional collaboration, empathy and learning, both in health professionals (25, 51) and in medical and nursing students (20, 21, 47, 53). Accordingly, the findings of this study provide a novel element in that they provide experimental evidence in favor of the predictive role that both empathy and learning have on the development of inter-professional collaboration. Based on these findings it is possible to attribute to empathy and learning an indirect influence on the improvement in subjective well-being (mediated by the improvement of inter-professional collaboration skills), that is in line with previous studies in which both elements have had some influence on the improvement of occupational health and well-being indicators attributed to them (27, 51, 54).

Finally, the analysis showed important differences in the development of inter-professional collaboration skills depending on the discipline and future professional direction. The findings indicated a greater development of inter-professional collaboration skills in nurses that is in line with what has been described in the extensive existing literature, in which these differences are attributed to the prevalence of socio-cultural models of hierarchical rather than complementary work, between medicine and nursing (21, 38, 51, 53, 55, 56). The analysis also showed differences in the development of inter-professional collaboration skills depending on the area in which the professionals wanted to specialize in their future work, being greater among those whose main interest was in the area of primary care. This higher score in inter-professional collaboration skills may be a consequence of there being a range of specialities with frequent contact with patients (such as primary care or pediatrics), in which the coordinated work between medical and nursing personnel is more intense, necessary and complementary. A fact that has been demonstrated in countries with a health system in which nursing has a more active role in primary care services (55, 57). Greater awareness of this fact could explain why professionals with an interest in directing their future work toward primary care showed a greater development of this skill.

In conclusion, the findings of this study provide solid evidence for the significant value of the prior development of three specific elements of professionalism: empathy with patients, lifelong learning skills and inter-professional collaboration skills, in improving the global perception of well-being (subjective well-being) of recently graduated professionals starting their first work experience in primary care services in rural communities. It also highlights the need to take measures that help reduce the psychological and emotional deterioration to which these young professionals are exposed in the last stages of their professional training and the importance of providing adequate support and personal and professional accompaniment during this first stage of their working life.

## Limitations

The study has performed a situational analysis of recently graduated health professionals when starting their first work experience. Therefore, the results of this work should be interpreted with some caution. A follow-up study of these professionals should be performed for the period of this rural medical service programme to assess the development of the elements measured over time and to assess the effect that this experience has on the development of their professional skill and their state of health and physical and emotional well-being.

## Data Availability Statement

The raw data supporting the conclusions of this article will be made available by the authors, without undue reservation.

## Ethics Statement

The studies involving human participants were reviewed and approved by Comité Ética de Investigación de La Rioja (CEICLAR). The patients/participants provided their written informed consent to participate in this study.

## Author Contributions

LV was in charge of the study's overall design. ERD, HLM, AOZ, and AVP carried out the study in the participating institution in Peru. LV and MSM statistically processed the data. LV and RD prepared the draft manuscript. All authors contributed to the present work, participated in the interpretation and processing of results, and reviewed and approved the final manuscript.

## Conflict of Interest

The authors declare that the research was conducted in the absence of any commercial or financial relationships that could be construed as a potential conflict of interest.
